# Do patients attempt and succeed in quitting smoking during tuberculosis treatment? A prospective cohort study

**DOI:** 10.1186/s12890-023-02693-0

**Published:** 2023-11-21

**Authors:** Jiwon Lee, Chaeuk Chung, Sung Soo Jung, Hye Kyeong Park, Sung-Soon Lee, Ki Man Lee, Jinsoo Min

**Affiliations:** 1 College of Medicine, The Catholic University of Korea, Seoul, Republic of Korea; 2 Division of Pulmonary and Critical Care Medicine, Department of Internal Medicine, Chungnam National University Hospital, Daejeon, Republic of Korea; 3 Division of Pulmonary and Critical Care Medicine, Department of Internal Medicine, Ilsan Paik Hospital, Inje University College of Medicine, Goyang, Republic of Korea; 4 Department of Internal Medicine, Chungbuk National University College of Medicine, Cheongju, Republic of Korea; 5 Division of Pulmonary & Critical Care Medicine, Department of Internal Medicine, Chungbuk National University Hospital, Cheongju, Republic of Korea; 6 Division of Pulmonary and Critical Care Medicine, Department of Internal Medicine, Seoul St. Mary’s Hospital, College of Medicine, The Catholic University of Korea, Seoul, Republic of Korea

**Keywords:** Pulmonary tuberculosis, Tobacco, Addiction, Cessation

## Abstract

**Background:**

Despite a well-known relation between smoking tobacco and the tuberculosis epidemic, the factors associated with smoking cessation in tuberculosis patients are unclear. This study aims to examine the cascade of smoking cessation and the factors associated with persistent smoking among tuberculosis patients.

**Methods:**

We conducted a prospective cohort study enrolling adult patients with pulmonary tuberculosis between 2016 and 2019 in the Republic of Korea. We examined the smoking status at baseline, followed the current smokers, re-examined their smoking status after 6 months of anti-tuberculosis treatment, and identified the factors associated with persistent smoking.

**Results:**

Of the 419 enrolled patients, 109 (26.0%) were current smokers at baseline. Of the 79 current smokers who completed the 6-month survey, 24 (30.4%) succeeded in quitting smoking after 6 months of treatment. The adjusted odds ratio for persistent smoking was 6.57 (95% confidence interval [CI], 1.76–27.83) for drinking and 0.15 (95% CI, 0.03–0.68) for diabetes comorbidity.

**Conclusion:**

Drinking alcohol and diabetes comorbidity were important factors in smoking cessation. Only one third of the tuberculosis patients in our study cohort succeeded in quitting smoking during the 6-month treatment period. More aggressive interventions for smoking cessation should be adopted within the national anti-tuberculosis program.

**Supplementary Information:**

The online version contains supplementary material available at 10.1186/s12890-023-02693-0.

## Background

It is estimated that *Mycobacterium tuberculosis* has infected 23% (1.7 billion) of the world’s population, resulting in 10 million new tuberculosis (TB) cases per year [1]. It is the leading cause of death from a single infectious pathogen, and in an effort to curb the global scourge, the World Health Organization (WHO) [2] has set tuberculosis as an important target in global health promotion. The relationship between tuberculosis and smoking tobacco has been well documented [3]: active smoking increases the risk of developing tuberculosis 2-2.5 times and is found to be significantly associated with poorer treatment outcome. Naturally, using effective smoking cessation interventions is an important factor in tuberculosis elimination. However, there is insufficient high-quality evidence assessing the effect of smoking cessation in improving tuberculosis treatment outcomes [4], largely owing to the heterogeneity of design, definition, and implementation of the individual researches [5]. Currently, it is difficult to identify the factors that are associated with smoking cessation in tuberculosis patients.

The Republic of Korea offers a unique study population in that it holds intermediate tuberculosis burden despite being a high-income country [6]. Also, smoking prevalence in Korea, especially among males (49.8%), is relatively high [7]. These circumstances offer a chance to assess the interplay of nicotine addiction, smoking cessation, tuberculosis symptoms, comorbidities, and other factors that could potentially impact the tuberculosis treatment outcome. While there are numerous studies assessing the association between smoking and tuberculosis, few focus on smoking cessation and the factors associated with it during anti-tuberculosis treatment. Thus, we conducted a prospective cohort study to examine the cascade of smoking cessation and the factors associated with persistent smoking among pulmonary tuberculosis patients.

## Methods

### Study design and participants

We enrolled adult patients with pulmonary tuberculosis from a prospective observational cohort study [8, 9] between November 2016 and September 2018 in Korea. This study was conducted at three university-affiliated hospitals, which participated in the public-private mix (PPM) project for tuberculosis control [10]. Tuberculosis specialist nurses in this project provided the education and support for the tuberculosis patients and monitored them for adherence to treatment. The following inclusion criteria were used: (1) age ≥ 19 years old, (2) diagnosed with or suspicious of pulmonary tuberculosis, and (3) treated with anti-tuberculosis medication for less than 1 month. Convenience sampling method was used to approach and recruit study participants. Participants were chosen from inpatients or outpatients based on their availability at that time. They were asked to participate in the cohort study, and informed consent forms were obtained.

### Data collection

Participants were evaluated upon study entry at each hospital for baseline characteristics. A case report form was used to prospectively collect demographical, clinical, and laboratory data from the patients. Nicotine dependence was assessed using the Fagerström test for nicotine dependence (FTND) instrument. FTND score 0–3 was defined as low nicotine addiction, 4–6 was defined as moderate nicotine addiction, and 7–10 was defined as high nicotine addiction [11, 12]. We categorized the current smokers into two groups, the lower addiction group (FTND 0–3) and the higher addiction group (FTND 4–10). After initiating anti-tuberculosis treatment, the participants were evaluated at 2-, 4-week, 2-, 4-, and 6-month visits. At the 6-month visit, a follow-up survey was conducted to re-evaluate tuberculosis-related symptoms and the present smoking status. Also, participants were checked for whether they had been offered advice on smoking cessation or had attempted to quit smoking during the anti-tuberculosis treatment. The tuberculosis specialist nurse informed the patients of the importance of smoking cessation during anti-tuberculosis treatment using the printed educational materials. Those who were interested in quitting smoking were referred to a smoking cessation support program sponsored by the National Health Insurance Service of Korea.

### Statistical analyses and sample size

Noncontinuous variables were presented as frequencies or percentages, and continuous variables were presented as means and standard deviations. The baseline characteristics of the participants were compared in univariable analyses, using the Chi-square test (or Fisher’s exact test) for noncontinuous variables and ANOVA (or Kruskal-Wallis) for continuous variables. Then, we performed additional univariable analysis to assess the association between the initial baseline characteristics and persistent smoking after 6 months of treatment. Subsequently, age, sex, and other clinical variables with *p* values < 0.05 were selected based on the univariable analysis and were further used to perform multivariable binary logistic regression in to evaluate the possible association between the variables and persistent smoking. Because it was hypothesized that high nicotine addiction could be associated with persistent smoking, the severity of nicotine addiction was also selected for the multivariable binary logistic regression model.

The variables of age, sex, nicotine addiction, drinking status, and diabetes comorbidity were included in the multivariable logistic regression model to identify the factors associated with persistent smoking after 6 months of anti-TB treatment. Because 5 variables were chosen for the analysis a priori, 50 events of persistent smoking were required to ensure a minimum of 10 events per variable [13]. Assuming that the proportion of persistent smoking at 6 months would be 60–70% [14], the minimum number of initial current smokers required to pool 50 events of persistent smoking after 6 months of anti-TB treatment was calculated to be between 72 and 84. Our multivariable logistic regression model analyzed 79 TB patients who completed the 6-month survey, a number adequate for identifying the factors linked to persistent smoking.

For a more comprehensive examination, we conducted another multivariable analysis that included only 3 variables of age, sex, and status of nicotine addiction. We also conducted another multivariable analysis that included the 5 variables plus dyspnea after 6 months of treatment, which was found to have a significant association in the univariable analysis. For the regression, a complete-case analysis approach was used, and unknown data was regarded as missing values. Results were accepted as statistically significant if *p* value < 0.05. All statistical analyses were performed using R Studio version 2022.12.0.

## Results

**Fig. 1:**
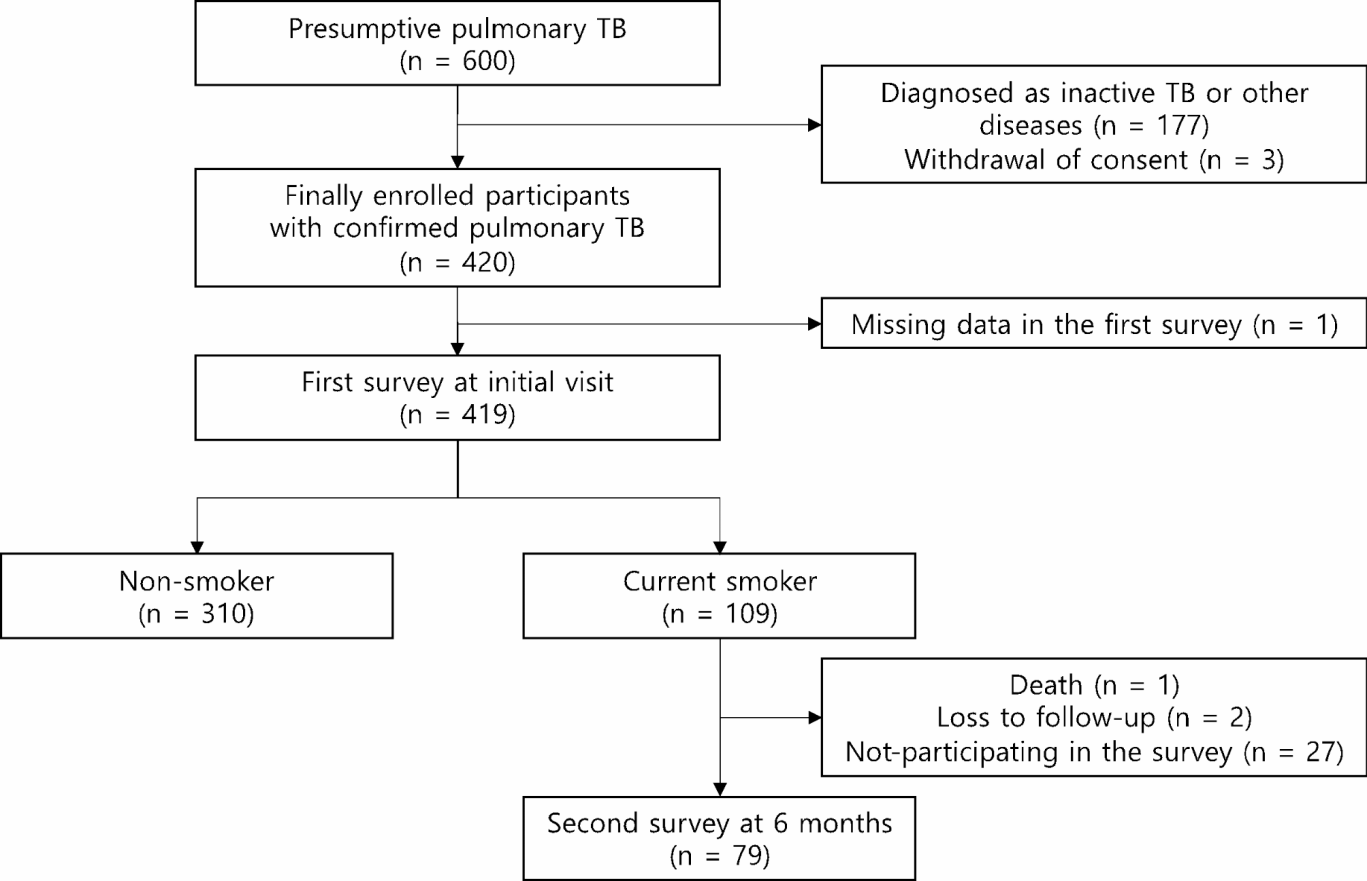
Flow chart of participant enrollment. TB, tuberculosis

After screening 600 patients with pulmonary tuberculosis, we finally enrolled and identified 109 (26.0%) current smokers and 310 (74.0%) non-smokers (Fig. 1). The baseline characteristics of the participants were compared across the non-smoker group, current smoker with lower addiction group, and current smoker with higher addiction group (Table 1). Compared to the non-smoking group or the lower addiction group, the higher addiction group showed higher male percentage (*p* < 0.05), tendency to be younger (*p* < 0.05), more frequent drinking habit (*p* < 0.05), greater tendency to have blue collar jobs than white collar jobs (*p* < 0.05), and more likely to have had prior tuberculosis history (*p* < 0.05). The higher addiction group reported more coughing symptoms initially compared to the lower addiction group (70.8% vs. 47.5%, *p* < 0.05). The clinical and laboratory characteristics of the participants were also compared across groups (Table S1). The higher addiction group showed higher pulse rate (*p* < 0.05) and higher body temperature (*p* < 0.05). In addition, the higher addiction group showed a higher level of inflammatory markers, such as white blood cell count (*p* < 0.05) and C-reactive protein (*p* < 0.05), and a lower level of albumin (*p* < 0.05).

**Table 1:** Baseline characteristics of the enrolled participants with pulmonary tuberculosis compared across groups with different current smoking status

Variables	All patients (n = 419)	Non-smoker (n = 310)	Current smoker		*p*-value
			Lower addiction (n = 61)	Higher addiction (n = 48)	
Male	257 (61.3)	157 (50.6)	54 (88.5)	46 (95.8)	< 0.050
Age < 65 years	236 (56.3)	146 (47.1)	49 (80.3)	41 (85.4)	< 0.050
Foreigner	17 (4.1)	12 (3.9)	2 (3.3)	3 (6.2)	0.647
Alcohol consumption	190 (45.3)	101 (32.6)	50 (82.0)	39 (81.2)	< 0.050
Occupation					< 0.050
White collar	53 (12.6)	36 (11.6)	11 (18.0)	6 (12.5)	
Blue collar	107 (25.5)	60 (19.4)	21 (34.4)	26 (54.2)	
Unemployed	259 (61.8)	214 (69.0)	29 (47.5)	16 (33.3)	
Comorbidities					
Diabetes mellitus	75 (17.9)	56 (1.81)	9 (1.73)	10 (2.17)	0.820
Chronic respiratory disease	30 (7.2)	26 (8.4)	2 (3.8)	2 (4.3)	0.354
Cancer	44 (10.5)	38 (12.3)	4 (7.4)	2 (4.3)	0.213
Prior tuberculosis history	83 (19.8)	49 (15.8)	17 (27.9)	17 (35.4)	< 0.050
Initial symptoms					
Cough	253 (60.4)	190 (61.3)	29 (47.5)	34 (70.8)	< 0.05
Dyspnea	88 (21.0)	68 (21.9)	9 (14.8)	11 (22.9)	0.427
Chest pain	54 (12.9)	34 (11.0)	12 (19.7)	8 (16.7)	0.127
Amount of current tobacco use (Packs-per-day)	0.80 ± 0.44		0.64 ± 0.31	0.99 ± 0.50	< 0.050
Intention to quit smoking					0.445
No plan to quit smoking	12 (11.0)		8 (13.1)	4 (8.3)	
In 1 month	62 (56.9)		37 (60.7)	25 (52.1)	
In 6 months	18 (16.5)		9 (14.8)	9 (18.8)	
After 6 months	17 (15.6)		7 (11.5)	10 (20.8)	

Values were expressed as number (percentage) or mean (± standard deviation)

**Fig. 2:**
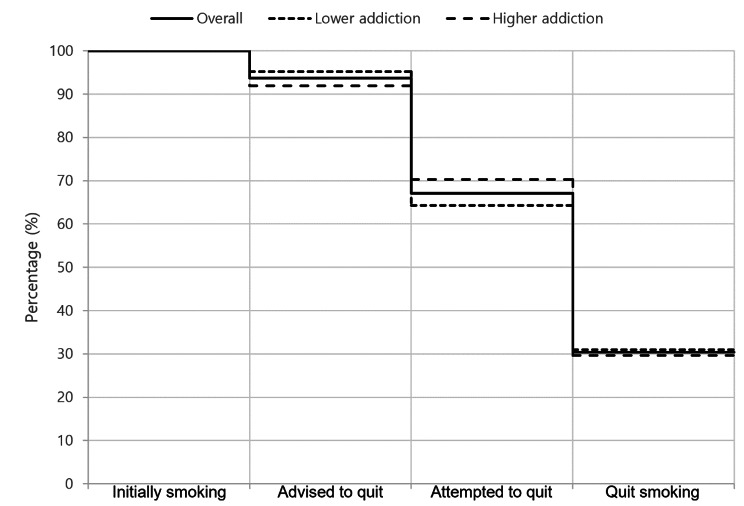
Cascade of smoking cessation among all the current smokers in different nicotine addiction groups. Values were expressed as number (percentage)

The cascade of smoking cessation of the participants who had initially been smoking at baseline were evaluated at the 6-month point (Fig. 2). Of the 109 current smokers at baseline, 30 did not completed the 6-month survey due to death (n = 1), loss to follow-up (n = 2), and not-participating in the survey (n = 27). Among the 79 current smokers who completed the 6-month survey, 74 (93.7%) had been advised by a healthcare professional to quit smoking during the treatment period, 53 (67.1%) had attempted to quit smoking during the treatment period, and 24 (30.4%) actually succeeded in quitting smoking by the 6-month point. The overall attempt to quit was higher in the higher addiction group, but not significantly (70.3% vs. 64.3%. *p* = 0.572). The overall successful smoking cessation was not different across groups (29.7% vs. 31.0%, *p* = 0.906). Out of the participants who attempted to quit, successful smoking cessation rate was higher in the lower addiction group, but not significantly (48.1% [13/27] vs. 42.3% [11/26], *p* = 0.906, results not shown). At the 6-month follow-up, the proportion of patients with dyspnea and chest pain in the higher addiction group was significantly higher, compared to the lower addiction group and the non-smoker group (20.0% vs. 4.1% vs. 7.0%, *p* < 0.050; 10.0% vs. 2.0% vs. 1.6%, *p* < 0.050) (Table S2).

Based on their smoking status at 6-month point, the participants who initially smoked at baseline were divided into the persistent smoking group and the successful quitting group (Table 2). Of the variables that were analyzed in the univariable analysis, drinking habit (90.9% vs. 62.5%, *p* < 0.05) and diabetes comorbidity (8.7% vs. 31.8%, *p* < 0.05) were found to be significantly different between the two groups. These variables were selected for the multivariable binary logistic regression. The adjusted odds ratios of persistent smoking were 6.57 (95% CI = 1.76–27.83, *p* < 0.05) for drinking and 0.15 (95% CI = 0.03–0.68, *p* < 0.05) for diabetes comorbidity (Table 3).

**Table 2:** Factors associated with persistent smoking at the 6-month follow-up visit among the initial current smokers

Characteristics	All patients (n = 79)	Persistent smoker (n = 55)	Smoking quitter (n = 24)	*p*-value
Nicotine dependence				0.906
Low	42 (53.2)	29 (52.7)	13 (54.2)	
Moderate/High	37 (46.8)	26 (47.3)	11 (45.8)	
Male	73 (92.4)	50 (90.9)	23 (95.8)	0.661
Age < 65 years	67 (84.8)	48 (87.3)	19 (79.2)	0.496
Drinking				< 0.05
Yes	65 (82.3)	50 (90.9)	15 (62.5)	
No	14 (17.7)	5 (9.1)	9 (37.5)	
Occupation				0.504
White collar	15 (19.0)	11 (20.0)	4 (16.7)	
Blue collar	35 (44.3)	22 (40.0)	13 (54.2)	
Unemployed	29 (36.7)	22 (40.0)	7 (29.2)	
Comorbidities				
Diabetes mellitus	11 (13.9)	4 (8.7)	7 (31.8)	< 0.05
Chronic respiratory diseases	2 (2.5)	0 (0.0)	2 (9.1)	0.090
Cancer	4 (5.1)	4 (8.5)	0 (0.0)	0.308
Prior tuberculosis history	22 (27.8)	14 (25.5)	8 (33.3)	0.472
Initial symptoms				
Cough	45 (57.0)	30 (54.5)	15 (62.5)	0.511
Dyspnea	15 (19.0)	11 (20.0)	4 (16.7)	1.000
Chest pain	16 (20.3)	8 (14.5)	8 (33.3)	0.072
Symptoms at 6 months				
Cough	14 (17.7)	11 (20.0)	3 (12.5)	0.533
Dyspnea	9 (11.4)	3 (5.5)	6 (25.0)	< 0.05
Chest pain	4 (5.1)	4 (7.3)	0 (0.0)	0.308
Microbiologic test results				
Positive AFB smear test result	20 (25.3)	12 (21.8)	8 (33.3)	0.279
Positive AFB culture test result	54 (68.4)	35 (63.6)	19 (79.2)	0.172
Resistant to either INH or RIF	9 (11.4)	7 (12.7)	2 (8.3)	0.715
Chest x-ray				
Bilateral disease on chest x-ray	20 (25.3)	15 (27.3)	5 (20.8)	0.545
Multi-lobar disease on chest x-ray ^1^	64 (90.1)	44 (89.8)	20 (90.9)	1.000

AFB, acid-fast bacilli; INH, isoniazid, RIF, rifampicin

Values were expressed as number (percentage) or mean (± standard deviation)

^1^ The total number was n = 71

**Table 3:** Multivariable analysis for factors associated with persistent smoking at the 6-month follow-up visit among initial current smokers

Variables	Model 1	Model 2	Model 3	Model 4
Moderate/High nicotine dependence	1.06 (0.40–2.81)	1.07 (0.40–2.92)	1.66 (0.54–5.49)	3.80 (0.99–19.42)
Age < 65 years		1.68 (0.44–6.08)	0.66 (0.12–2.95)	0.49 (0.07–2.53)
Male		0.47 (0.02–3.28)	0.72 (0.03–5.36)	0.49 (0.02–3.88)
Drinking			6.57 (1.76–27.83)	6.00 (1.45–28.80)
Diabetes mellitus			0.15 (0.03–0.68)	0.09 (0.01–0.47)
Dyspnea at 6 months				0.07 (0.01–0.44)

Values are expressed as odds ratio and 95% confidence interval. Model 1 is the result of univariable logistic regression analysis. Models 2, 3 and 4 are the results of univariable logistic regression analysis

## Discussion

This is the first study of this size that examines tuberculosis patient characteristics including nicotine dependence, exploring the variables that influence persistent smoking after 6-month of anti-tuberculosis treatment. The higher addiction group showed higher male percentage, tendency to be younger, more frequent drinking habit, greater tendency to have blue collar jobs than white collar jobs, and higher inflammatory reactions. The participants who initially smoked at baseline were questioned on their smoking status at 6-month point, and we compared the cascade of smoking cessation across the higher and lower addiction groups, though no significant difference was discovered across groups. Based on the multivariable logistic regression analysis, drinking was found to increase the odds of persistent smoking, whereas diabetes comorbidity was found to decrease the odds of persistent smoking. Smoking plays an important role in determining tuberculosis outcome, and understanding the factors that influence smoking in tuberculosis patients can be helpful to healthcare professionals in guiding patients to quit smoking and respond better to treatment.

The initial smoking rate (26.0%) was higher than the known smoking rate in general population in South Korea (19.3% [15]). This study attempted to determine what factors affect persistent smoking in tuberculosis patients. Unlike what was predicted, nicotine addiction as assessed by the FTND does not affect persistent smoking in tuberculosis patients. As shown in the smoking cessation cascade, the successful quitting rate was equally low in both groups, despite the high rate of being advised on quitting. Some studies report the effectiveness of simple advice from physicians in encouraging patients to quit smoking [16, 17], but this study shows that it is not effective enough in tuberculosis patients. Because smoking is associated with worse treatment outcomes including mortality in tuberculosis patients [18], smoking should be more strictly discouraged in this patient group. Our results also imply that even in a tuberculosis patient group with an already a high rate of being advised on quitting, a more active anti-smoking campaign should be launched, for patients with low or high addiction levels alike. Such an active campaign could include intensive, multidisciplinary, holistic approach that exposes the patient to more continuous, professional management [19]. Other than using hospital-based medical resources, the patients could be reached via digital devices and healthcare platforms [20].

Although nicotine dependence as assessed by the FNTD was unexpectedly found not to affect persistent smoking at 6-month point in our study, a few other variables were shown to be associated with persistent smoking, such as drinking habit and diabetes comorbidity. As expected, drinking is positively associated with persistent smoking, aligning with the previous studies that report the association between drinking and smoking [21, 22] and the synergistic interplay of substance abuse and addictive behavior [23]. This implies that in order to effectively treat tuberculosis, more intensive management of drinking problems is necessary.

As a surprise, diabetes comorbidity is negatively associated with persistent smoking in tuberculosis patients. Although many studies have explored the impact of smoking on the diabetes disease burden [24–26], no studies have examined the effect of diabetes comorbidity on smoking behavior, especially in tuberculosis patients. This discovery may be reflective of how diabetic patients are constantly exposed to medical professionals, especially in South Korea where healthcare is easily accessible under universal health coverage. Due to the chronic nature of diabetes and the necessity of frequent visits to healthcare facilities, diabetic patients in South Korea are kept under continuous surveillance in medical systems, providing them with greater medical insight and greater exposure to advice from medical professionals. This could have been an important factor encouraging the tuberculosis patients with diabetes to quit smoking and make necessary lifestyle modifications. Other studies have already emphasized the crucial role of continual support and professional treatment in implementing and maintaining lifestyle changes in diabetic patients [27]. The same strategy could be translated into encouraging smoking tuberculosis patients to quit smoking, through more continual support and higher exposure to professional advice.

The WHO’s End Tuberculosis Strategy emphasizes a patient-centered approach in tuberculosis treatment in order to promote adherence, improve the quality of life, and relieve suffering of the patients [28]. This approach focuses not only on medical treatment, but also on supporting people to overcome their socio-economic, cultural, legal, and psychological difficulties. Healthcare providers are encouraged to understand the tuberculosis patients’ needs and concerns, recognizing the barriers in diagnosing and treating the disease. Tuberculosis patients are known to have factors conducive to failing to quit smoking, such as low socioeconomic status [29]. It is important to construct interactive collaborations between the national anti-tuberculosis and anti-tobacco programs, within a single healthcare system. The anti-tuberculosis programs and the smoking cessation programs should organize health resources together to collaborate in encouraging tuberculosis patients to quit smoking. Healthcare organizations need to incorporate smoking cessation into their tuberculosis control programs and promote tobacco-free lifestyles. Healthcare providers need to learn about current smoking cessation campaign and be able to educate patients on the harms of smoking [30]. Local community resources, public-private partnerships, and mobile and digital healthcare platforms are some examples of the tools that can be used. Successful engagement of these tools could lead to greater compliance and decreased losses during follow-up. This will eventually lead to higher smoking cessation rates and improved tuberculosis treatment outcomes, lowering the public burden of this disease. Future qualitative and quantitative studies could explore strategies for an effective anti-smoking program in tuberculosis patients.

The major strength of the present study is its systematic approach to data collection. Data was collected prospectively by trained tuberculosis specialist nurses as part of a large prospective cohort study. It is the first study of this size that explores nicotine dependence using FTND in tuberculosis patients, with follow-up at multiple points. It revealed a previously veiled association between diabetes comorbidity in tuberculosis patients and higher smoking cessation rate at 6 months, which hints at a potential strategy for smoking cessation in tuberculosis patients.

However, this study also has some limitations. First, a larger sample size might have yielded a greater power to detect differences across the groups. Second, there might have been some selection bias precisely because it was conducted in university-affiliated hospitals actively participating in PPM projects, recruiting more severe tuberculosis patients on referral from primary healthcare facilities. Third, there is the possibility of selection bias because convenience sampling strategy was used in this study, and its non-probabilistic nature leads to reduced generalizability.

## Conclusions

This study has explored nicotine dependency and the variables that influence smoking cessation in tuberculosis patients. Only one third of the tuberculosis patients in our study cohort succeeded in quitting smoking by the end of the 6-month tuberculosis treatment period. This reflects the necessity of a more effective strategy for smoking cessation. Although the overall smoking cessation rate was not significantly affected by nicotine dependency, drinking was positively associated with persistent smoking, while diabetes comorbidity was negatively associated with persistent smoking. This suggests that a more intensive management of the patient’s drinking problems is necessary to improve tuberculosis treatment outcome. Continual support and professional treatment (like what an average diabetic patient experiences in South Korea) could be an important strategic approach in implementing and maintaining lifestyle changes in tuberculosis patients. Smoking cessation interventions should be part of a patient-centered approach in treating tuberculosis patients (as recommended by the WHO), and anti-tobacco policy needs to be incorporated into national anti-tuberculosis programs.

### Electronic supplementary material

Below is the link to the electronic supplementary material.

Supplementary Material 1

## Data Availability

The ownership of the primary datasets lies with the Korea Disease Control and Prevention Agency. The datasets generated and/or analysed during the current study are available from the corresponding author on reasonable request with permission of the Korea Disease Control and Prevention Agency. The corresponding author should initially be contacted for the request accessing the raw data.

## List of abbreviations

WHO: World Health Organization
TB: Tuberculosis
COSMOTB: cohort study of pulmonary tuberculosis
AFB: Acid-fast bacilli
NAAT: nucleic acid amplification test
CT: computed tomography
