# Supplementary material for: Do patients attempt and succeed in quitting smoking during tuberculosis treatment? A prospective cohort study
Source: BMC Pulm Med. 2023 Nov 21;23:456. doi: 10.1186/s12890-023-02693-0 (PMC10664422; doi:10.1186/s12890-023-02693-0)
Supplement: Supplementary file 1 — Supplementary Material 1 [file 12890_2023_2693_MOESM1_ESM.docx]

**Table S1. Clinical and laboratory characteristics of the enrolled participants with pulmonary tuberculosis compared across groups with different current smoking status**

| Characteristics | All patients  (n = 419) | Non-smoker  (n=310) | Current smoker | | *p*-value |
| --- | --- | --- | --- | --- | --- |
|  |  |  | Lower addiction  (n = 61) | Higher addiction  (n = 48) |  |
| Physical examination |  |  |  |  |  |
| Pulse rate | 84.5±14.8 | 84.3 (±15.1) | 81.7 (±14.1) | 89.9 (±12.7) | <0.05 |
| Body temperature | 36.82±0.57 | 36.78 (±0.52) | 36.75 (±0.55) | 37.12 (±0.80) | <0.05 |
| Body mass index (kg/m^2^) ^1^ | 21.39±3.12 | 21.51 (±3.12) | 21.45 (±3.11) | 20.58 (±3.04) | 0.152 |
| Microbiological testing |  |  |  |  |  |
| Positive AFB smear test result | 116 (27.7) | 88 (28.4) | 15 (24.6) | 13 (27.1) | 0.828 |
| Positive AFB culture test result | 287 (68.5) | 213 (68.7) | 39 (63.9) | 35 (72.9) | 0.598 |
| Resistant to either INH or RIF | 40 (9.5) | 25 (8.1) | 7 (11.5) | 8 (16.7) | 0.144 |
| Bilateral disease on chest x-ray | 92 (22.0) | 66 (21.3) | 12 (19.7) | 14 (29.2) | 0.423 |
| Nutritional markers |  |  |  |  |  |
| Protein (g/dL) | 6.76±0.72 | 6.72±0.70 | 6.94±0.59 | 6.77±0.92 | <0.05 |
| Albumin (g/dL) | 3.76±0.70 | 3.71±0.67 | 4.11±0.66 | 3.69±0.81 | <0.05 |
| BUN (mg/dL) | 13.68±6.70 | 14.02±7.04 | 12.67±5.34 | 12.78±5.77 | 0.219 |
| Hemoglobin (g/dL) |  |  |  |  |  |
| Male ^2^ | 13.28±2.04 | 12.93 (±1.93) | 13.98 (±1.73) | 13.66 (±2.46) | <0.05 |
| Female ^2^ | 11.96±1.42 | 11.94 (±1.41) | 12.49 (±1.75) | 12.15 (±1.20) | 0.486 |
| Inflammatory marker |  |  |  |  |  |
| White blood cell (/μL) ^3^ | 7651±2964 | 7494 (±2975) | 7607 (±2384) | 8711 (±3360) | <0.05 |
| Neutrophil (%) ^3^ | 66.7±12.4 | 67.1 (±12.7) | 63.8 (±11.4) | 67.8 (±11.5) | 0.095 |
| Lymphocyte (%) ^3^ | 22.3±11.1 | 22.1 (±11.3) | 24.4 (±9.7) | 20.7 (±11.0) | 0.104 |
| Platelet (x1000/μL) ^3^ | 281±110 | 283 (±109) | 265(±103) | 285 (±124) | 0.687 |
| C-reactive protein (mg/dL) ^4^ | 3.39±5.06 | 3.53 (±5.35) | 1.97 (±3.21) | 4.08 (±4.62) | <0.05 |

AFB, acid-fast bacilli; INH, isoniazid, RIF, rifampicin

Values were expressed as number (percentage) or mean (± standard deviation).

^1^ The total number was n=413.

^2^ The total number was n=405 for male and female group combined, n=248 for male and n=157 for female.

^3^ The total number was n=405.

^4^ The total number was n=364.

**Table S2. Secondary survey at the 6-month follow-up visit among 79 current smokers compared to non-smokers**

| Characteristics | All patients  (n = 345) | Non-smoker  (n=256) | Current smoker | | *p*-value |
| --- | --- | --- | --- | --- | --- |
|  |  |  | Lower addiction  (n = 49) | Higher addiction  (n = 40) |  |
| Symptoms at 6-month visit |  |  |  |  |  |
| Cough | 54 (15.7) | 39 (15.2) | 7 (14.3) | 8 (20.0) | 0.713 |
| Dyspnea | 28 (8.1) | 18 (7.0) | 2 (4.1) | 8 (20.0) | <0.05 |
| Chest pain | 9 (2.6) | 4 (1.6) | 1 (2.0) | 4 (10.0) | <0.05 |
| Increase in body mass index at 6-month visit ^1^ | 0.36±1.37 | 0.25±1.39 | 0.31±1.08 | 1.03±1.40 | <0.05 |

Values were expressed as number (percentage) or mean (± standard deviation).

^1^ The total number was n=307.
